# Advanced NiCr/NiSi Thin-Film Thermocouples for Precise Temperature Sensing in Lithium-Ion Battery Systems

**DOI:** 10.3390/s25113438

**Published:** 2025-05-30

**Authors:** Xiyao Liu, Yanpeng Mao

**Affiliations:** National Engineering Laboratory of Coal-Fired Pollutants Emission Reduction, School of Energy and Power Engineering, Shandong University, Jinan 250100, China; 202200180042@mail.sdu.edu.cn

**Keywords:** NiCr/NiSi thin-film thermocouples, lithium-ion batteries, thermal management internal, temperature measurement, battery safety

## Abstract

Efficient thermal management is critical for the performance, safety, and longevity of lithium-ion batteries, particularly in new energy vehicles. This paper presents the development and application of a NiCr/NiSi thin-film thermocouple fabricated via magnetron sputtering on a polyimide substrate, aiming to provide high-precision, fast-response internal temperature measurements for lithium-ion battery systems. The thermocouple demonstrates a Seebeck coefficient of approximately 40.95 μV/°C and a repeatability error of only 0.45%, making it highly suitable for capturing transient thermal events. The main innovation of this work lies in the comprehensive integration of simulation and experimental validation to optimize the thermocouple’s performance for lithium-ion battery applications. This includes static calibration, external short-circuit, and puncture tests, which collectively confirm the thermocouple’s reliability and accuracy. Additionally, the study explores the impact of ambient temperature variations on internal battery temperatures, revealing a nearly linear increase in internal temperature with rising ambient conditions. The findings offer valuable insights for improving battery thermal management systems, establishing early warning thresholds for thermal runaway, and enhancing the overall safety of lithium-ion battery applications.

## 1. Introduction

Amid growing environmental concerns, particularly regarding fossil fuel combustion in the road transportation sector—which accounts for 61% of total CO_2_ emissions—manufacturing industries worldwide are increasingly focusing on the development of new energy vehicles that utilize low-cost energy sources [1,2,3,4]. However, temperatures that are too high or too low can affect the performance of the Li-ion ternary battery case [5,6,7]. Therefore, a battery thermal management system is critical to maintain battery temperatures within the proper range and to reduce the overall temperature difference between batteries [8,9]. Temperature sensors play a leading role in battery thermal management systems [10,11].

This study develops a method for measuring temperature using a thin-film thermocouple, which exhibits high sensitivity due to its thin electrode layers of different materials and fast response resulting from its thin thermal contacts [12,13,14]. It has a temperature measurement range of −200 to 1800 °C. Previous research has explored various thin-film thermocouple configurations for high-temperature applications. Yang Ke et al. [15] prepared ITO/Pt thin-film thermocouples on a nickel-based high-temperature alloy substrate to measure the temperature of hot components in a gas turbine engine. The results showed that the internal electron flow of the thermocouple is inhibited at high temperatures, resulting in a significant decrease in the Seebeck coefficient. Yan Liu [16] prepared a metal-semiconductor (WRe26–In_2_O_3_)-type probe thin-film thermocouple using a microelectromechanical systems process. The results indicated that the measurement performance of the thermocouple is unstable at high temperatures (above 620 °C). Hao Xie [17] prepared a platinum–rhodium thermocouple on an alumina ceramic substrate. The results demonstrated that the cooling rate of the thermocouple during the cooling stage is slow, causing the measured temperature to be higher than the actual temperature.

Additionally, Werschmoeller et al. [18] prepared a tungsten–rhenium thin-film thermocouple 300 μm from the PCBN cutting edge to detect transient temperatures near the tip of the tool. The results showed that the sensitivity of the thin-film thermocouple is very low, only 8.87 μV/°C in the temperature range of 20–125 °C. Radajewski et al. [19] used Al_2_O_3_ tube-covered thermocouples to detect temperature in a graphite tool. The results indicated that the measured temperature of the thermocouple in the transient temperature measurement process lags behind the actual temperature. Kim et al. [20] fabricated thermocouples in the shape of flexible fibers using thermoelectric graphene composite fibers. The results showed that the thermocouple can only operate effectively from room temperature to 70 °C.

Resistive sensors (10.3390/s20133652) are highly sensitive and diodes (10.1109/TED.2020.2999391) are low-cost and simple in structure but both are susceptible to temperature [21,22], while thin-film thermocouples are resistant to high temperatures and have a fast response time [23,24,25], making them more suitable for measuring the temperature of ternary lithium-ion battery cases.

This paper adopts a combination of simulation and experimental methods. First, the optimal solution C for NMC (Nickel Manganese Cobalt) battery packs is derived through simulation. Subsequently, experiments involving external short circuits, pin-pricks, and varying ambient temperatures are conducted to develop a thermal runaway warning scheme. These experiments also confirm the reliability, accuracy, and reproducibility of the NiCr/NiSi thin-film thermocouple temperature measurement tool developed in this study.

## 2. Thin-Film Thermocouple Design and Fabrication

**Figure 1:**
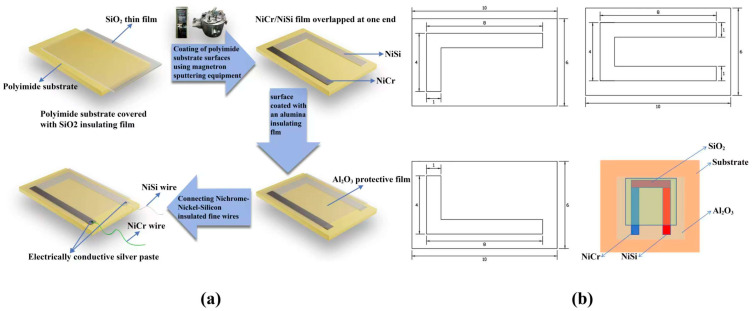
(**a**) Fabrication process of homemade NiCr/NiSi thin film thermocouple, and (**b**) schematic of mask plate and homemade NiCr/NiSi thin film thermocouple.

In this investigation, NiCr/NiSi thin film thermocouples were produced on polyimide substrates using magnetron sputtering with a thickness of 1500 nm, as illustrated in Figure 1a. To prevent thermoelectric potential loss and ensure electrical isolation of the thermocouple from the metal substrate, an insulating thin film of Al_2_O_3_ was prepared on the substrate in an oxygen atmosphere using an aluminum target with a purity of 99.99% as a magnetron sputtering source via radio frequency magnetron sputtering. Next, NiSi and NiCr targets (Chino New Material (Beijing) Technology Co., Ltd, Beijing, China), each with 99.99% purity, were used as the magnetron sputtering source and the direct current (DC) magnetron sputtering technique was employed under the cover of a mask plate as shown in Figure 1b, and ultimately, L-shaped NiCr and NiSi thin films as shown in Figure 1b were sputtered and deposited on the Al_2_O_3_ insulating layer. Finally, in order to avoid oxidation, abrasion, and damage during the cutting process, SiO_2_ protective films were prepared on NiSi and NiCr thin films in an oxygen atmosphere using a silicon target with a purity of 99.99% as a magnetron sputtering source by the radio frequency magnetron sputtering technique. 

## 3. Static Calibration of Thin Film Thermocouples

### 3.1. Thin Film Thermocouple Characterization

**Figure 2:**
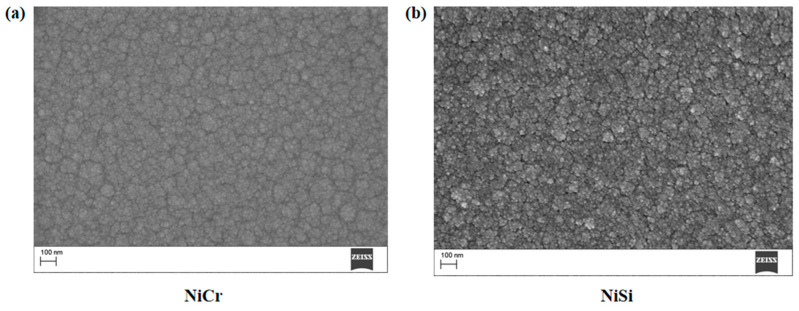
(**a**) SEM images of NiCr thin film thermocouples, and (**b**) SEM images of NiSi thin film thermocouples.

Figure 2a presents SEM images of the NiCr thin film, illustrating a uniform distribution of particles with consistent size and morphology, which suggests good material homogeneity during the fabrication process. The surface exhibits smoothness, potentially contributing to reduced surface defects and enhanced mechanical stability. Additionally, the film demonstrates a dense microstructure indicative of high material compactness. Figure 2b shows the SEM image of the NiSi thin film, characterized by a complex microstructure with varied particle size and shape distributions. The surface morphology reflects the intrinsic material features, while the microstructural arrangement provides insights into the film’s structural characteristics.

### 3.2. Build an Experimental Platform

The temperature measurement system consists of an oven (Guangdong Grantham Group Co., Ltd, Foshan City, China), compensation wire (Jiangsu Jiangrun Copper Co., Ltd, Yixing City, China), beaker filled with ice water, SW605A (Meichuan Automation Instrument Factory in Hangzhou, China) and DT9205A (Meichuan Automation Instrument Factory in Hangzhou, China).

**Figure 3:**
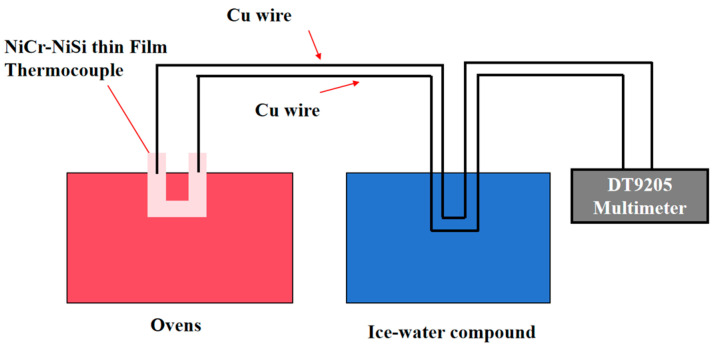
Static calibration experiment platform.

In this experiment, an SW605A infrared temperature gun was used to measure the oil temperature at the hot end. Having a built-in program, a temperature measurement range of 50~200 °C, a temperature error of ±0.15 °C, and the option to select the thirteen-point localization of the temperature measurement mode are just a few of the features of the SW605 infrared temperature gun. This helps to make the measurement data more reliable and minimize the measurement error from the system. Using room temperature as the temperature reference simplifies the experimental process. The DT9205A digital multimeter is a 4-digital multimeter with a minimum resolution of 1 μV in DC voltage measurement, and the specific static calibration system is shown in Figure 3. The homemade and standard NiCr-NiSi silk film thermocouples were both placed in a beaker and calibrated using the above static calibration system. Standard NiCr/NiSi silk thermocouple has a comparative role; if the standard NiCr/NiSi silk thermocouple Seebeck coefficient and homemade NiCr/NiSi film thermocouple Seebeck coefficient do not have a large difference, this can be explained by the fact that the homemade NiCr/NiSi film thermocouple is well prepared, with excellent performance. The cold end of the thermocouple was connected to the positive and negative terminals of the multimeter through NiCrNiSi compensating leads as shown in Figure 3. At the same time, the actual temperature measurements of the homemade NiCrNiSi film thermocouple and the standard NiCrNiSi wire film thermocouple were recorded with the multimeter until the multimeter reached a steady state.

The goals of static calibration are as follows: it increases the accuracy of the thin film thermocouples, prepares them for the upcoming temperature measurement calculations, and characterizes the Seebeck coefficient of the thermoelectric and high-temperature resistance characteristics [26,27,28]. The standard K-type thermocouple and the experimentally prepared NiCr/NiSi thin-film thermocouple’s hot ends are both placed in the same temperature field during the static calibration test, with the cold ends’ temperature maintained at 0 °C. A multimeter is used to record the thermopotentials between the cold and hot ends of the standard K-type thermocouple and the experimentally prepared NiCr-NiSi thin-film thermocouple. Finally, the thermopotentials between the cold and hot ends of the experimentally prepared NiCr-NiSi thin-film thermocouple and the traditional K-type thermocouple are mapped using the experimental data. Plotting the NiSi thin film thermocouple’s thermopotential vs. temperature characteristic curve from the experimental data yields the Seebeck coefficient. This value is then contrasted with that of the conventional K-type thermocouple.

### 3.3. Repeatability and Consistency

#### 3.3.1. Consistency Test

**Figure 4:**
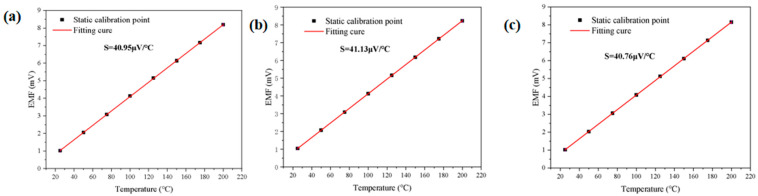
(**a**) The first set of static calibration experimental data, (**b**) the second set of static calibration experimental data, and (**c**) the third set of static calibration experimental data.

Finally, the experimental data were used to plot the thermoelectric potentials between the cold and hot ends of the NiCr–NiSi thin-film thermocouple and the standard K-type thermocouple. By analyzing the thermoelectric potential versus temperature characteristic curve of the NiCr–NiSi thin-film thermocouple, the Seebeck coefficient was calculated and compared to that of the standard K-type thermocouple. The results are presented in Figure 4.

#### 3.3.2. Repeatable Experiments

To assess the repeatability of the homemade thin-film thermocouple, the same device was repeatedly heated from 26.8 °C to a steady state across five test cycles. During each cycle, the electromotive force was recorded and subsequently fitted, with the results presented in Figure 4.

As illustrated in Figure 4, the three measurements of the homemade thin-film thermocouples exhibit close agreement. Specifically, the Seebeck coefficient of the homemade NiCr–NiSi thin-film thermocouple was determined to be 40.95 μV/°C in the first static calibration test, 41.13 μV/°C in the second, and 40.76 μV/°C in the third. Repeatability error analysis of the data:

(1) $$\sigma =\frac{\sqrt{{\left(x_{i}-x\right)}^{2}}}{n-1}$$

In the formula, σ—standard deviation; $x_{i}$—Seebeck’s coefficient for test group i; x—average Seebeck coefficient; n—actual number of tests.

Repeatability error:

(2) $$\delta =\frac{\sigma }{x}\times 100\%=0.45\%$$

In the formula, σ—standard deviation; x—mean Seebeck coefficient; δ—repeatability error.

The error formula was utilized to assess the experimental data, revealing that the maximum repeatability error for the same homemade thin-film thermocouple was 0.45% within the specified heating range. These findings demonstrate that the NiCr/NiSi thin-film thermocouple developed in this study exhibits excellent repeatability and is suitable for determining the internal temperature of onboard lithium-ion battery cases. Furthermore, the calibration method R^2^ values were 0.99996, 0.99999, and 0.99998, respectively.

### 3.4. Dynamic Calibration

**Figure 5:**
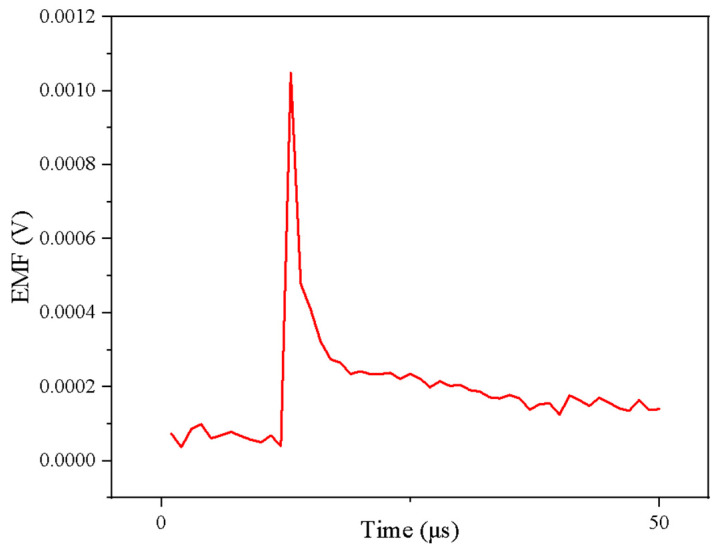
Dynamic calibration results.

A laser model LR-DTQ-532 was used, with a pulse width of 8.325 ns in the nanosecond range, which is extremely small compared to the microsecond response time of a thin film thermocouple, making it a qualified excitation source. The results are shown in Figure 5.

## 4. Temperature Measurement

### 4.1. Emulate

COMSOL Multiphysics 6.2 software was employed to determine the optimal topology of the ternary lithium-ion battery box. The results indicate that the open-circuit voltage error during charging and discharging should not exceed 0.025 V. Moreover, there is no significant difference between the simulation results obtained after 5 min and those after 4 h of operation, suggesting that shorter simulation times are sufficient. Additionally, the number of charge–discharge cycles should be limited to 350 to avoid capacity degradation. These findings contribute to a reduction in the duration of subsequent experimental procedures.

#### 4.1.1. Determining the Topology of the Lithium-Ion Battery Box for the Experiments in This Paper Using Simulation

**Figure 6:**
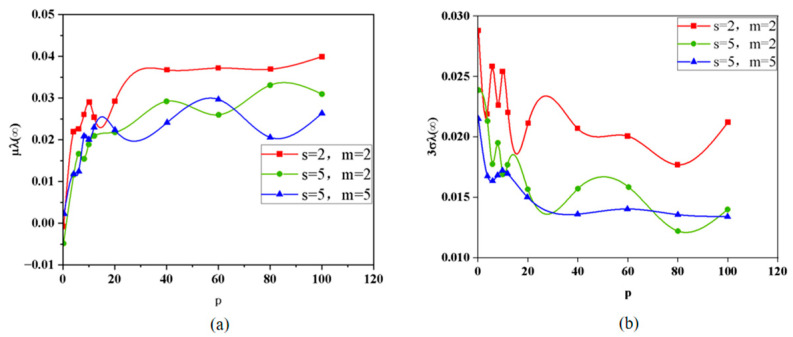
(**a**) Curve fitting results for the mean value of the current distribution coefficient of the battery with different topologies, and (**b**) fitting results of 3σ for different topologies.

The topology of ternary lithium-ion battery packs plays a critical role in determining both their performance and safety. In this study, we investigate the effects of the total number of cells in series (s), the number of battery modules (m), and the number of individual cells per module (p) on the average value and maximum error (denoted as 3σ) of the battery current distribution coefficient through simulation. As shown in the simulation results in Figure 6a,b, the battery current distribution coefficient increases rapidly at first and then more gradually as p increases, depending on different values of s and m. Specifically, for a given s and p, a smaller m results in a higher average current distribution coefficient and a larger 3σ. Similarly, for a fixed m and p, a smaller s also leads to a higher current distribution coefficient and 3σ. In all cases, increasing p tends to increase the average current distribution coefficient while reducing 3σ. Based on these observations, the topology configuration of s = 2, m = 2, and p = 100 is selected for the experimental phase of this study.

#### 4.1.2. Probability Density Profile of Parallel Battery Pack Capacity

**Figure 7:**
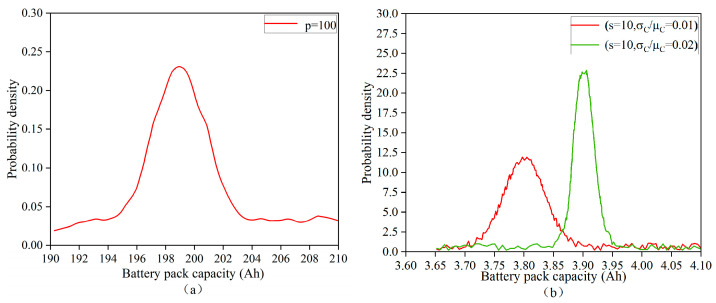
(**a**) Monte Carlo simulation results of battery pack capacity for the number of parallel battery cells p = 100, and (**b**) Monte Carlo simulation results for different σc/μc battery pack capacities (s = 10).

A total of 10^4^ simulations were conducted using the Monte Carlo method, with the parameters set as s = 2, p = 100, and m = 2. The resulting probability density function (PDF) curves of the battery pack capacity are shown in Figure 7a. The simulation results indicate that the initial capacities of the battery cells follow a normal (Gaussian) distribution. The probability density reaches its peak value of 0.25 when the battery capacity is 199 Ah, with a corresponding standard deviation set to 10^−2^.

#### 4.1.3. Determine the Optimal Solution for the NMC Battery Pack C

**Figure 8:**
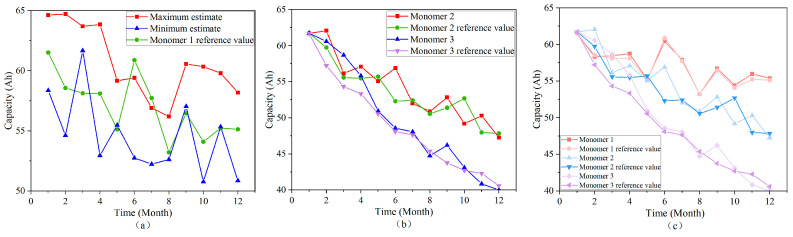
(**a**) Impact of estimation error of SOC on the results of Monomer 1 capacity estimation, (**b**) typical solution for Monomer 1 directly used in the estimation results for Monomer 2 and Monomer 3 (optimal solution C), and (**c**) NMC cell estimation results based on single segment charging voltage segments.

In this study, numerical simulations were performed using COMSOL to evaluate the fluctuations in the maximum, minimum, and reference capacity values of Monomer 1 over time. As shown in the simulation results in Figure 8a, all three values—maximum estimate, minimum estimate, and reference—exhibit a similar trend: a rapid decline within the first 0–2 months, followed by stabilization, and then another sharp decrease after 3 months. Throughout the simulation period, the reference capacity of Monomer 1 consistently remains between the maximum and minimum estimates, never exceeding these bounds.

The typical solution derived for Monomer 1 was directly applied to Monomers 2 and 3, with the corresponding simulation results shown in Figure 8b. The capacities of Monomer 2 and Monomer 3, along with their respective reference values, both exhibit a rapid decline over time. This demonstrates the feasibility of applying the typical solution in terms of capacity degradation trends, as the actual capacities of Monomers 2 and 3 closely track their reference values. Based on this consistency, the optimal solution, referred to as Solution C, was identified.

Subsequently, an NMC cell was simulated using this optimal Solution C, with the results presented in Figure 8c. The overall trend reveals a significant reduction in capacity over time, with the actual capacity values of each monomer closely aligning with their reference values. This confirms the suitability of Solution C for application in NMC battery systems.

**Figure 9:**
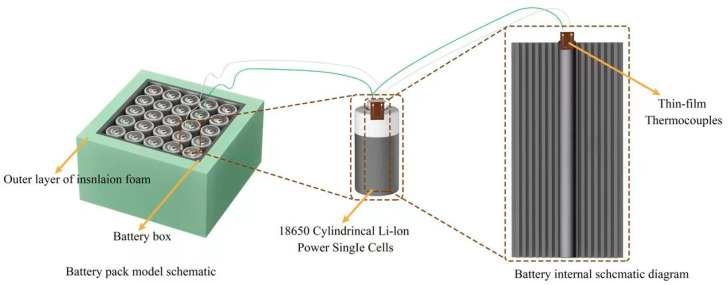
Schematic diagram of temperature measurement simulation of ternary lithium battery.

To further evaluate the predictive accuracy of the simulation results, a set of ternary lithium-ion battery pack models was designed based on the optimized simulation parameters. These models, intended for experimental validation, are schematically illustrated in Figure 9.

#### 4.1.4. Absolute Errors in Predicting Open-Circuit Voltage of Batteries During Charging and Discharging by Three Methods

**Figure 10:**
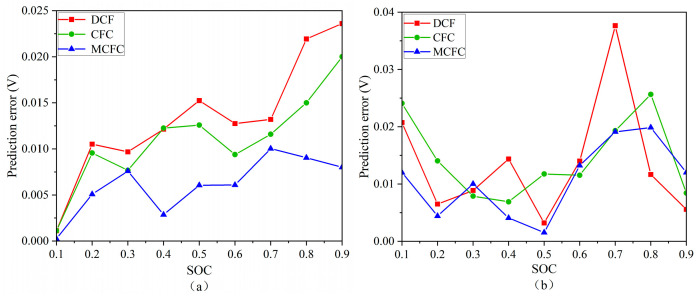
(**a**) Absolute error in predicting battery open-circuit voltage for the three methods–charging process, and (**b**) absolute error in the prediction of battery open-circuit voltage for the three methods–discharge process.

In this study, three methods—Discounted Cash Flow (DCF), Closed-form Continuous-time Neural Networks (CFC), and Molten Carbonate Fuel Cell (MCFC)—are employed to predict the absolute error of the open-circuit voltage. These methods are used to validate the accuracy of voltage-related data obtained in subsequent experiments, based on numerical simulations performed in COMSOL during the charging and discharging of lithium-ion batteries at various states of charge (SOCs) [29,30]. The simulation results are presented in Figure 10a,b.

During the charging process, the absolute error in the open-circuit voltage exhibits an overall upward trend across all three methods, ranging from 0 to 0.025 V as the SOC increases from 0.1 to 0.9. Similarly, during the discharging process, the absolute error ranges from 0 to 0.04 V within the same SOC range.

When using the DCF method for charging prediction, the error initially increases, fluctuates slightly, and then rises rapidly as SOC increases. The CFC and MCFC methods both show fluctuating trends followed by a sharp increase in error. In contrast, the SOC-based method shows only minor fluctuations and generally tends toward zero.

For discharging predictions using the DCF method, the error initially decreases with increasing SOC, reaching 0 V at 20% SOC. After a period of slight fluctuation, a sudden increase occurs at 60% and 70% SOC, peaking at 0.009 V, before abruptly dropping back to 0 V. When using the CFC method, the prediction error starts at 0 V, increases sharply at 20% SOC, reaches a peak of 0.002 V at 40% SOC, then drops to 0 V before rising again. The MCFC method shows some variation in prediction error as SOC increases, but the error eventually converges to zero.

These results indicate that all three methods can reliably capture the open-circuit voltage behavior of lithium-ion batteries under different SOCs, with small absolute errors that support the validity of the experimental data.

#### 4.1.5. Exploring the Feasibility of Reducing Voltage Test Time with Simulation

**Figure 11:**
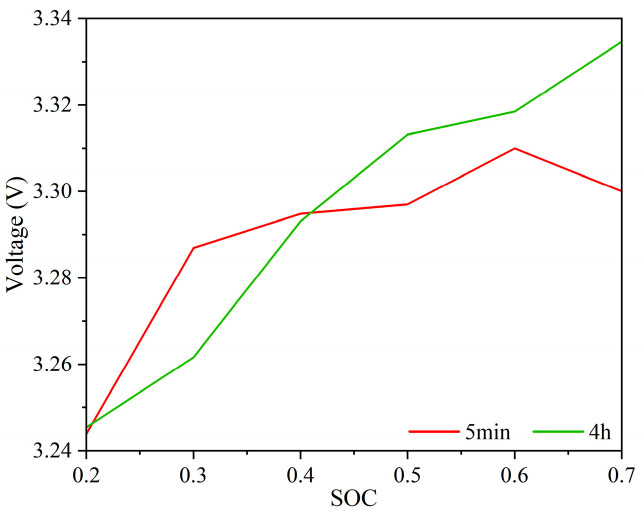
OCV-SOC curve of LPF cell.

The simulation results, presented in Figure 11, investigate the impact of test duration on voltage measurements at various states of charge (SOCs), aiming to enhance experimental efficiency. The data show that the ternary lithium battery exhibits both five-minute and four-hour voltage profiles as SOC increases. During this period, the voltage initially rises rapidly, then stabilizes, followed by a slower increase.

At SOC levels between 20% and 40%, the voltage measured after five minutes is slightly lower than that measured after four hours. Conversely, at SOC levels between 50% and 70%, the five-minute voltage readings are slightly higher than the four-hour results. For other SOC values, the voltage measurements under both durations are nearly identical.

These observations suggest that test duration has minimal influence on the voltage results across different SOCs. Therefore, in subsequent experiments, once the voltage has stabilized, extended testing times will be deemed unnecessary and thus omitted to improve overall experimental efficiency.

#### 4.1.6. Influence of Battery Cell Capacity on Battery Pack Capacity

To investigate the influence of cell capacity inconsistency on battery pack performance, two ratios—σc/μc = 0.01 and σc/μc = 0.02—were selected for simulation at series configurations of s = 10 and s = 100, respectively. As illustrated in the simulation results shown in Figure 7b, for the case of s = 10, the scenario with σc/μc = 0.01 shows a more rapid rise in probability density compared to σc/μc = 0.02, reaching a peak density of 9 at a battery pack capacity of 3.9 Ah. After this peak, the probability density begins to decline. Conversely, the σc/μc = 0.02 case starts to rise after this point and reaches a higher peak probability density of 17.5 at a slightly lower capacity of 3.88 Ah, after which it also declines rapidly to zero.

These results indicate that, when σc is held constant, an increase in the σc/μc ratio leads to a decrease in the expected capacity of the battery pack and an increase in the standard deviation. This highlights that greater inconsistency in the initial cell capacities results in a lower average pack capacity and greater variability. Therefore, in this study, the individual cell capacities within the battery pack are strictly controlled to be uniform in order to minimize systematic errors and enhance overall reliability.

#### 4.1.7. Simulating the Capacity Degradation Process of a Battery Pack

**Figure 12:**
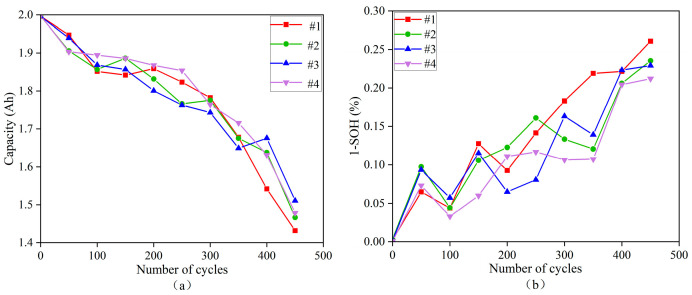
(**a**) Cell individual capacity degradation dataset in battery packs (cell capacity degradation dataset), and (**b**) cell individual capacity degradation dataset in battery packs (converted cell SOH curves).

A battery pack consisting of four parallel-connected cells was used to validate the effectiveness of the proposed method. The capacity degradation process of the pack was simulated using the Alawa toolkit for battery degradation modeling, with the parameters of each individual cell listed in Table 1. As illustrated in the simulation results shown in Figure 12a,b, the capacities of all four types of cells gradually decline as the number of charge–discharge cycles increases. However, a sharp drop in capacity is observed once the number of cycles reaches 350. Therefore, it is recommended that the experimental cycle count not exceed 350 in order to avoid significant degradation and to maintain the reliability of the test data.

#### 4.1.8. Investigation of Electrochemical Stability of Battery Packs

**Figure 13:**
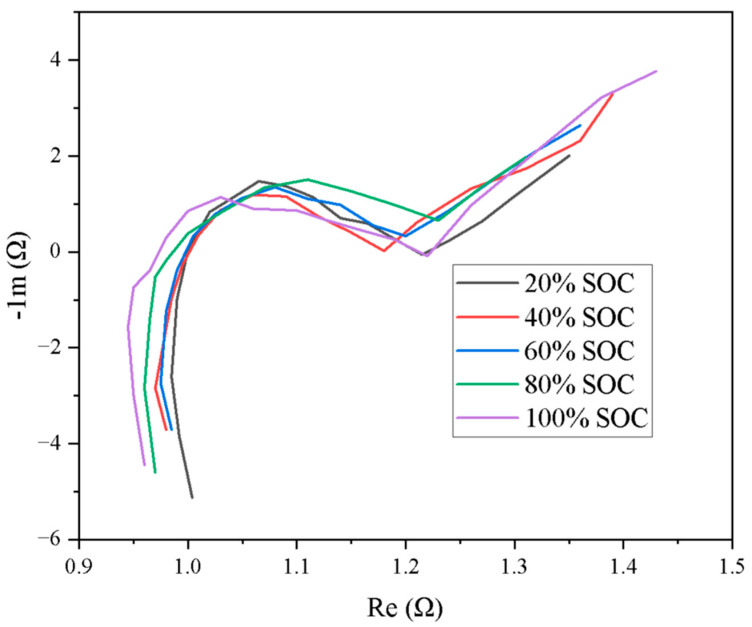
Schematic diagram of Nissan Leaf 2011 battery module MD27EIS.

To validate the approach presented in this study, two sets of electrochemical impedance spectroscopy (EIS) tests were conducted on each of the 26 battery module datasets from the 2011 Nissan Leaf. The impedance spectra were measured across a frequency range of 0.001 Hz to 1 kHz, resulting in a total of 130 EIS datasets. These tests were performed at 20% state-of-charge (SOC) intervals to evaluate variations in EIS characteristics under different SOC conditions. As shown in the simulation results in Figure 13, the impedance curves at various SOC levels exhibit an initial rapid rise, followed by a plateau, and then another sharp increase. The results indicate that the electrochemical behavior of the battery modules tends to stabilize within the frequency range of 1.05 × 10^−3^ Hz to 1.27 × 10^−3^ Hz. In contrast, outside this range—particularly in the absence of data around 1.27 × 10^−3^ Hz—the electrochemical characteristics appear unstable.

### 4.2. Experiments

A representative single lithium-ion battery within the battery pack was selected through the simulation results, and a thin-film thermocouple embedded in the battery was used to obtain the temperature during the experiment. To get a better picture of the temperature rise of the battery pack and to simulate the heat dissipation inside the battery pack of an electric vehicle, insulation foam was arranged around the battery pack to provide thermal insulation.

#### 4.2.1. Construction of the Overall Experimental Platform

The battery selected for testing was positioned in the center of the battery pack. The thin-film thermocouple sensor used for temperature measurement has a range of −20 °C to 200 °C with an accuracy of ±0.1 °C, which adequately covers the operating temperature range of the battery. The FuelCon lithium battery test platform was utilized for testing, and a multifunctional multimeter was used to collect the temperature and voltage data measured by the thermocouples [31,32,33,34,35]. The battery under test was a ternary lithium-ion battery.

#### 4.2.2. Abuse Behavior of Lithium Batteries with Different States of Charge During External Short Circuit

Based on the modeling results, a representative lithium-ion battery cell from within the battery pack was selected for temperature measurement. During the experiment, a thin-film thermocouple was inserted into the battery to monitor its temperature. To better understand the temperature rise within the battery pack and simulate the heat dissipation conditions of an electric vehicle’s battery pack, insulation foam was wrapped around the battery pack to provide thermal insulation.

##### Temperature Change Rule During External Short Circuit of Different SOC Batteries

**Figure 14:**
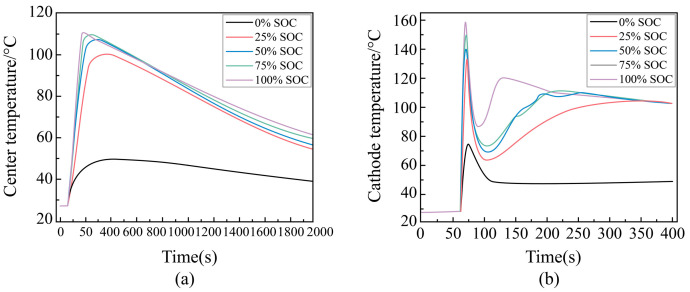

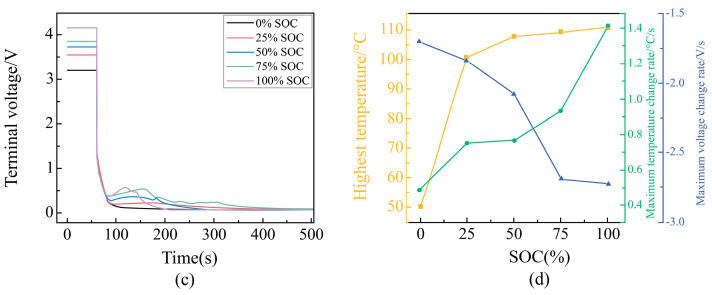
(**a**) Center temperature during external shorting of different SOC circuits, (**b**) negative terminal temperature during external short circuit for different SOC cells, (**c**) voltage variation during external short circuit for different SOC cells, and (**d**) variation rule of characteristic parameters with SOC during external short circuit of single cell.

To investigate the temperature change patterns of lithium batteries with various states of charge (SOC) during an external short circuit, this study focuses on the temperature trends at the center of the battery cells. The results are presented in Figure 14a. It is evident that during an external short circuit, the center temperatures of Li-ion ternary batteries at different SOCs initially increase and then decrease. Additionally, the center temperature rise is proportional to the SOC during the short circuit event. The maximum temperature of the cell at 0% SOC is lower than that at 25% SOC, reaching only 49.9 °C, which aligns with the temperature of the negative electrode. However, there is not a significant difference in the center temperatures between the 25% SOC cell (maximum temperature: 101.3 °C) and the 100% SOC cell (maximum temperature: 111.5 °C). Notably, the center temperature at 100% SOC rises to 116 °C within 180 s of an external short circuit. Given these findings, it is recommended that the lithium-ion battery case be designed with a rated maximum temperature of 116 °C to prevent potential accidents.

##### Maximum Temperature During External Short-Circuit for Different SOC Cells

The analysis of the experiments in the previous section indicates that the negative electrode temperature is the highest during the external short circuit of a single-cell battery. Therefore, this study first examines the changes in the negative electrode temperature during the external short circuit of batteries at five different states of charge (SOC), 0%, 25%, 50%, 75%, and 100%, under the same ambient conditions over time, as shown in Figure 14b. The results demonstrate that as SOC decreases, the maximum temperature of the negative terminal during the external short circuit also decreases for each cell.

Batteries with 25% to 100% SOC exhibit a similar pattern, with the negative electrode temperature initially increasing then decreasing, followed by another increase and subsequent decrease. In contrast, the negative terminal temperature of the 0% SOC battery rises to its peak and then begins to fall, likely due to the limited stored power in the battery and the excessively high short-circuit current, leading to a rapid depletion of the stored energy.

The results show that at 100% SOC, the negative electrode temperature surges to 158 °C after an external short circuit. To prevent potential accidents, it is recommended that the lithium-ion battery case be designed to withstand a maximum temperature of 158 °C.

##### Voltage Change Rule During External Short Circuit of Different SOC Batteries

The voltage change pattern during the external short circuit of batteries at different states of charge (SOC) is shown in Figure 14c. Initially, the battery voltage across different SOCs is stable. After 70 s, as the external short circuit begins, the voltage of batteries at different SOC levels rapidly decreases to below 1 V. The voltages drop sharply from 4.25 V (100% SOC), 3.8 V (75% SOC), 3.48 V (50% SOC), 3.27 V (25% SOC), and 3.15 V (0% SOC) to less than 1V. This is followed by a slight rebound in voltage, eventually converging to 0 V.

Notably, the voltage change behavior of the 0% SOC battery differs from that of other batteries; after the rapid voltage drop, there is no rebound, and the voltage continues to decrease steadily. The results indicate that an external short circuit in the battery causes a sudden drop in terminal voltage to below 1 V. Consequently, during an external short circuit, the battery can experience a rapid power loss, and as the final voltage drops to 0 V, the battery becomes completely ineffective. This can lead to the sudden breakdown of the new energy vehicle, potentially resulting in traffic accidents. Therefore, it is crucial to detect whether the battery is experiencing an external short circuit when a sudden drop in terminal voltage is observed to avoid such dangers.

##### Change Rule of Characteristic Parameters During External Short Circuit of Different SOC Batteries

To establish a database for battery warning systems, this study selects the maximum temperature, maximum rate of temperature change, and maximum rate of voltage change as characteristic parameters during the external short circuit of a single battery. The variation patterns of these parameters are illustrated in Figure 14d. As the SOC of the battery increases, the maximum temperature initially rises rapidly, then more gradually, reaching a peak value of 111.2 °C. The maximum rate of temperature change also initially increases, remaining constant from 25% SOC until 50% SOC, where it rises sharply again to a maximum value of 1.42 °C/s.

Conversely, the maximum rate of voltage change decreases gradually with increasing SOC. At 50% SOC, this rate starts to decrease rapidly until 75% SOC, after which it slows down again, reaching a minimum value of −2.53 V/s. Overall, the results show that the maximum temperature and maximum rate of temperature change generally increase with SOC, whereas the maximum rate of voltage change shows a decreasing trend as SOC increases. Additionally, during an external short circuit, as the SOC decreases, there are sudden drops and rises in both the maximum rate of temperature change and the maximum rate of voltage change.

Given these findings, it is important to check for potential external short-circuit failures in lithium-ion ternary batteries when relevant changes are observed. This is crucial to prevent battery pack explosions due to external short circuits, which pose a significant safety risk.

##### Temperature Change Rule of Each Point with Time During External Short Circuit of Battery Module

**Figure 15:**
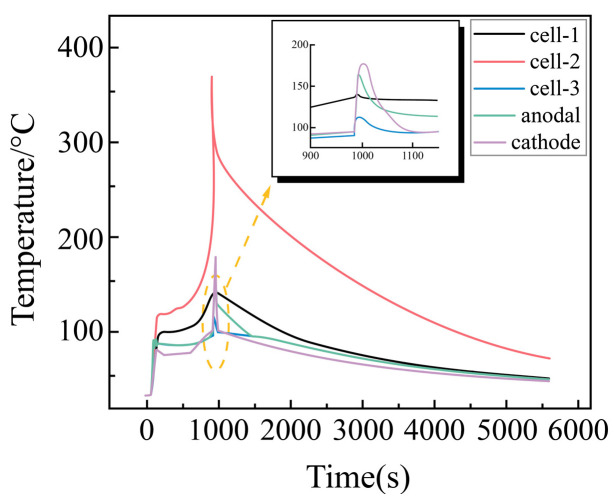
Temperature variation at each point during external short circuit of battery module (with local magnification).

In this study, we investigated the temperature change patterns during an external short circuit in a battery module consisting of Cell-1, Cell-2 (intermediate cell), and Cell-3, along with the temperatures at the two pole lugs. The results are presented in Figure 15. In order to get a clearer picture of the temperature change, the temperature change at the short-circuit instant at the temperature measurement area except Cell-2 is enlarged in Figure 15. It is observed that the temperature of the pole lugs of each cell and the battery module rises rapidly to approximately 100 °C at the moment of short circuit. Among these, Cell-2 reaches the highest temperature, significantly exceeding the temperatures of the positive and negative lugs of the other cells and the module, followed by Cell-1 and Cell-3. The negative lug temperature of the battery module is slightly higher than the positive lug temperature, consistent with the pattern observed in the external short circuit of a single battery.

As the temperature continues to rise, there is a sudden and rapid increase in the temperature of the negative electrode lug of the battery module, coinciding with the thermal runaway of Cell-2. However, the other cells do not experience thermal runaway and only exhibit a transient temperature increase. After the thermal runaway of Cell-2, the temperature of the battery module gradually decreases with no observed rebound, and no thermal runaway occurs in the remaining cells. These findings indicate that to prevent thermal runaway, it is crucial to effectively monitor the temperature changes in the intermediate cell and the negative electrode.

#### 4.2.3. Abuse Performance of Lithium Batteries with Different States of Charge During Needling

Since the safety status of the battery is also affected by the sudden impact contingency that occurs when the vehicle is in motion, this paper also experimentally analyzes the abuse performance of batteries with different states of charge during pinprick as well.

##### Abuse Behavior of Lithium Batteries in the Same State of Charge at Various Points During Pinning

**Figure 16:**
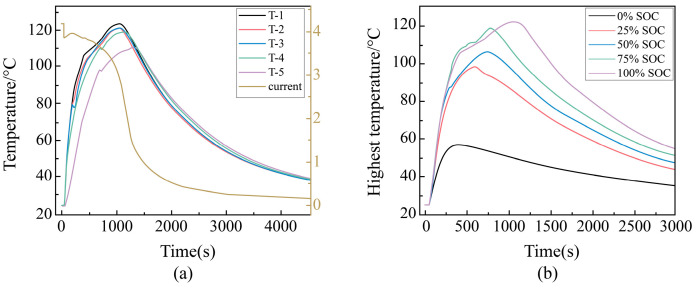

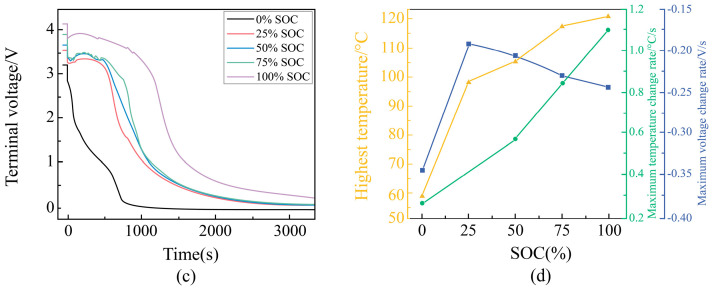
(**a**) Temperature and voltage changes during pinning of 100% SOC cells, (**b**) trend of maximum temperature during pinning for different SOC cells, (**c**) terminal voltage variation during pinning for different SOC cells, and (**d**) variation rule of characteristic parameters with SOC during pinning of single cell.

Five temperature measurement points were established at 100% SOC: T-1 (located below the stinger), T-2 (on the left side of the stinger), T-3 (on the right side of the stinger), T-4 (at the center of the cell), and T-5 (on the upper side of the cell). The terminal voltage was also measured simultaneously, with the results presented in Figure 16a. At the moment of penetration, the battery voltage drops rapidly from 4.17 V to 3.86 V. The voltage then slightly recovers, rising to 3.93 V before continuing to decline, with the rate of decline slowing gradually until the voltage reaches zero.

Among the temperature measurement points, T-1, located below the stinger, exhibits the highest temperature, reaching 119.5 °C. The temperatures at T-2 and T-3, located on the left and right sides of the stinger, are similar but slightly lower than T-1. T-4, positioned at the center of the cell, follows in temperature, while T-5, on the upper side of the cell, has the lowest temperature, being lower than T-4.

The results indicate that the temperature trends at each point of the cell during penetration are essentially the same, with the voltage dropping to zero after a period, rendering the cell inoperable.

##### Changes of Maximum Temperature During Needling Outside Different SOC Cells

In this research, we examined the temperature change at the T-1 point in batteries at five different states of charge (SOC): 0%, 25%, 50%, 75%, and 100%. The results are shown in Figure 16b. The analysis from the previous section indicates that the temperature change at each position of a single cell during the pinning phase is generally consistent.

As the SOC increases, the maximum temperature at the T-1 measurement point on the underside of the felting needle during penetration initially rises and then falls more slowly, maintaining a higher maximum temperature for a longer duration. The maximum temperature reached at 25% SOC (97 °C) was not significantly different from that at 100% SOC (122 °C). However, the temperature of the 0% SOC battery is considerably lower than that of the 25% SOC battery. This may be due to the limited capacity of the 0% SOC battery, which leads to rapid depletion of stored energy. The results indicate that at 100% SOC, the temperature at T-1 surges to 122 °C at 1050 s after pinning. To prevent accidents, the lithium-ion battery case should be designed to withstand a maximum temperature of 122 °C during penetration events.

##### Voltage Change Pattern During External Pinning of Different SOC Cells

The voltage change patterns for batteries at different states of charge (SOC) during pinning are shown in Figure 16c. The voltage change trends for batteries with 25%, 50%, and 75% SOC are essentially the same. At the onset of the external short circuit, the terminal voltage of the 75% SOC battery is slightly higher than that of the 50% SOC battery, which in turn is slightly higher than that of the 25% SOC battery. However, after 1230 s, the voltage trends of these three SOC levels converge and continue to gradually decline until the terminal voltage approaches 0 V.

For the 100% SOC battery, although its terminal voltage is initially much higher than that of the 75% SOC battery, the overall change trend is consistent with the other three SOC levels. In contrast, the voltage change pattern of the 0% SOC battery differs from the others. After pinning, there is no voltage recovery phase; instead, it directly enters a stage of rapid decline. This behavior may be attributed to the limited amount of stored power in the 0% SOC battery and the rapid generation of high current following pinning, leading to a swift depletion of the remaining energy. According to the results, a battery that experiences pinpricking will cause a sudden drop in terminal voltage. In other words, during the pinprick period, the new energy vehicle may experience brief underpowering. If the final voltage drops to zero, the battery will become completely ineffective, which could result in a sudden breakdown and traffic accident. For this reason, it is important to determine whether the battery is pinpricked when a sudden drop in terminal voltage occurs in order to prevent potential hazards.

##### Changes of Characteristic Parameters During Needling of Different SOC Cells

To establish the database required for battery warning systems, this study selects the maximum temperature, the maximum rate of temperature change, and the maximum rate of voltage change as characteristic parameters during the pinning of a single battery. The variation trends of these parameters are shown in Figure 16d. As the state of charge (SOC) of the battery increases, the maximum temperature rises rapidly until 25% SOC and then gradually reaches 122 °C. Concurrently, the maximum rate of temperature change increases sharply, reaching a peak value of 1.09 °C/s.

The maximum rate of voltage change initially increases rapidly with SOC until 25% SOC, after which it decreases slowly, reaching a minimum value of −0.24 V/s. The results indicate that a rapid decline in the maximum rate of temperature change, a significant drop in maximum temperature, or a steep decrease in the rate of voltage change should prompt an investigation into potential pinning failure of the ternary lithium battery. This is crucial for preventing hazardous events, such as battery box explosions, which pose significant safety risks.

##### Mass Change Before and After Cell Needling

**Figure 17:**
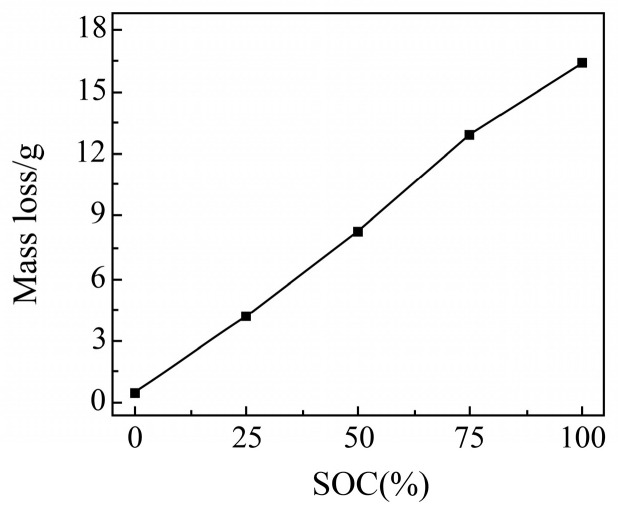
Mass change after cell pinning.

No significant expansion was observed during the needling process. To investigate the gas production of cells at different states of charge (SOC) during needling, the changes in the mass of the cells before and after the needling were compared, as shown in Figure 17. The results indicate that there is minimal mass loss in 0% SOC cells (only 0.53 g). However, as the SOC increases, the mass loss of the cells after needling also increases, following an approximately linear trend. Notably, the highest mass loss of 16.35 g was observed in 100% SOC cells. These findings suggest that the battery generates a significant amount of gas during needling, and both the decomposition of battery materials and gas production increase linearly with SOC. Therefore, it is important to avoid needling the battery at high SOC to prevent the risk of explosion due to excessive gas production.

##### Change Rule of Temperature Change Rate with Time at Each Point During Pinning of Battery Module

**Figure 18:**
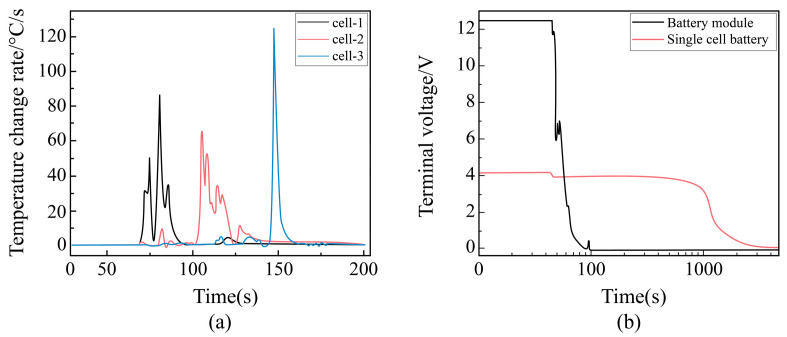
(**a**) Rate of change of temperature of each cell during cell module pinning, and (**b**) terminal voltage change during cell module pinning vs. single cell.

In this study, the temperature measurements for Cell-1, Cell-2 (intermediate cell), and Cell-3 within a battery module composed of these three cells are presented in Figure 18a. The temperature change rate of Cell-1 increases first, followed by Cell-2. After a period of fluctuation and decline, the temperature change rate of Cell-3 suddenly spikes to 127 °C/s before rapidly decreasing. Eventually, the temperature change rates of Cell-1, Cell-2, and Cell-3 all approach zero. These results indicate that needling causes a rapid change in temperature rates, which diminishes progressively from the nearest cell to the farthest. This suggests that the rate of temperature change decreases as the distance from the needling point increases, leading to a corresponding drop in temperature.

##### Voltage Change Law with Time at Each Point During Needling

The voltage change trend of the battery module during the pre-pinning period is similar to that of a single cell, with both experiencing partial voltage drops, as shown in Figure 18b, which compares the terminal voltage changes between the single cell and the battery module during pinning. Following the initial voltage drop, the single cell displays a prolonged plateau period before slowly declining. In contrast, the battery module’s voltage drops rapidly to zero after a very brief period of stable fluctuations. This rapid drop may be attributed to the higher voltage of the battery module, which results in a higher current and a faster rate of heat generation. This, in turn, can cause the internal diaphragm to collapse rapidly, leading to a significant internal short circuit. As the battery module undergoes thermal runaway, the charged active material inside is quickly consumed, causing a rapid voltage decline until it eventually reaches zero. These findings suggest that battery modules are more susceptible to thermal runaway during pinning than single cells. Therefore, it is crucial to appropriately reduce the voltage of the battery module and continuously monitor its temperature changes to mitigate the associated risks.

### 4.3. Influence of Ambient Temperature on the Temperature Variation of the Battery

**Figure 19:**
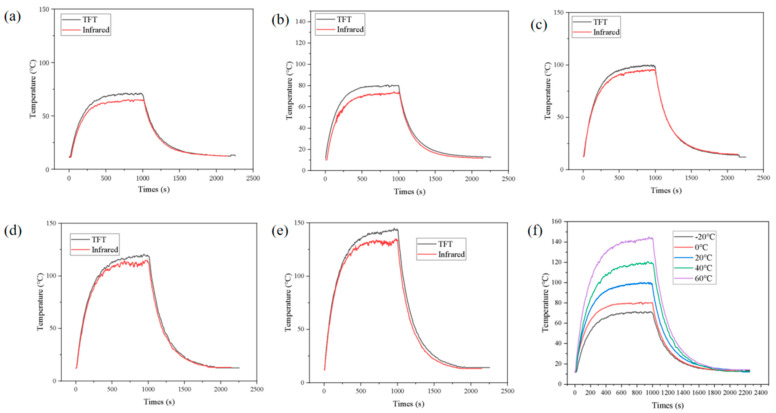
Temperature change rule of ternary lithium battery under different ambient temperatures. (**a**–**e**) Temperature measurement results at ambient temperatures of −20, 0, 20, 40, and 60°C. (**f**) Comparison of Thin Film Thermocouple Results.

Figure 19a–e: IR vs. TFT. In Figure 19a–e, the temperature rises rapidly in the initial stage, peaks and then gradually decreases and stabilizes. This trend indicates that the battery heats up quickly in the initial stage, and then the heat is gradually dissipated. The temperature trends of IR (black curve) and TFT (red curve) are basically the same, verifying the reliability of the two measurement methods. However, the peak value of the IR curve is slightly higher than that of the TFT curve, which may be due to the slight interference of the IR measurement by environmental factors, while the results of the thin-film thermocouple are more accurate

At lower ambient temperatures (e.g., Figure 19a,b), the temperature peaks are lower and the rate of decrease is slower. This suggests that the heat dissipation from the cell is slower at low temperatures, which may be due to the low absorption capacity of heat at low temperatures. At higher ambient temperatures (e.g., Figure 19d,e), the temperature peaks are higher and the rate of increase is faster. This may be due to more intense chemical reactions within the battery at high ambient temperatures, resulting in faster heat generation.

Figure 19f shows the TFT measurements at different ambient temperatures. As the ambient temperature increases (from −20 °C to 60 °C), the temperature peak increases significantly. At −20 °C, the temperature peak is 71.4 °C, while at 60 °C, the temperature peak is above 140 °C and reaches 145.5 °C. This indicates that the ambient temperature has a significant effect on the heating behavior of the battery. This change may be related to the chemical reaction rate inside the battery. At high ambient temperatures, the chemical reaction inside the battery is more intense, resulting in faster and greater heat generation. At higher ambient temperatures (e.g., 40 °C and 60 °C), the rate of temperature rise is faster, suggesting that heat is more likely to accumulate at high ambient temperatures. At lower ambient temperatures (e.g., −20 °C and 0 °C), the rate of temperature rise is slower, which may be due to the fact that heat is dissipated more quickly at lower temperatures. At all ambient temperatures, the temperature declined gradually after reaching its peak, but at higher ambient temperatures (e.g., 40 °C and 60 °C), the rate of decline was faster. This may be due to the fact that at high temperatures, heat is more easily dissipated into the surrounding environment. At low temperatures (e.g., −20 °C and 0 °C), the rate of temperature decrease is slower, indicating that heat is more difficult to dissipate at low temperatures.

## 5. Conclusions

1. Introduction and validation of high-precision temperature measurement using NiCr/NiSi thin-film thermocouples:

This paper presents the use of NiCr/NiSi thin-film thermocouples for temperature measurement in batteries, with experimental results confirming their high reliability and repeatability. The experimental data indicate that the Seebeck coefficient of the thin-film thermocouple is 40.95 μV/°C, with a deviation of only 0.95 μV/°C from the standard NiCr/NiSi wire thermocouple, demonstrating excellent measurement precision. Furthermore, the maximum repeatability error observed in repeated tests was merely 0.45%, confirming the suitability of this thermocouple for battery thermal management systems. This innovation addresses the limitations of traditional temperature measurement methods, which often fail to accurately capture transient temperature changes, thereby significantly enhancing accuracy and response speed in temperature monitoring.

2. Optimization of battery pack topology through simulation, enhancing safety and reducing experimental time:

Using numerical simulations, this paper derives an optimal battery pack topology (s = 2, m = 2, p = 100), which was found to enhance experimental safety. Simulation results indicate that the maximum open-circuit voltage measurement error does not exceed 0.025 V, and the voltage change results after 5 min and 4 h are nearly identical, suggesting that the experiment duration can be effectively shortened. This approach improves both the safety and efficiency of the experiments while reducing system error through strict control of cell capacity consistency in the battery pack design.

3. Detailed safety warning data from external short-circuit and puncture experiments:

4. Comparing the temperature results of IR thermometer and TFT, the results show that the temperature results of TFT are always higher than those of the IR thermometer, which is because TFT can measure the temperature of the Li-ion battery more directly and avoid interference from the environment. We took measurements at different temperatures (−20, 0, 20, 40, 60 °C)

5. The temperature results of the Li-ion battery at −20, 0, 20, 40, and 60 °C were measured by TFT, and the results showed that the maximum working temperature of the Li-ion battery at −20 °C was 71.4 °C and that at 60 °C was 145.5 °C, which proved the capability of Li-ion battery to work in low temperatures.

This study quantifies temperature and voltage variations during external short-circuit and puncture conditions for the first time. The data indicate that the maximum negative electrode temperature can reach 158 °C during a short circuit at 100% state of charge (SOC) and 122 °C during puncture. These findings establish critical threshold values for thermal runaway and explosion risks, offering valuable guidance for designing safer battery protection mechanisms. Additionally, the study introduces temperature and voltage variation rates as monitoring indicators, providing essential data support for lithium battery safety warning systems.

## Figures and Tables

**Table 1:** Parameters of single cell.

Battery Model	$\sigma_{H}^{2}$	θ	$R_{V}$	$\mu_{\lambda ,0}$	${\sigma^{2}}_{\lambda ,0}$
#1	0.0257	1.142	0.012	0.1543	1.3050 × 10^−4^
#2	0.0324	1.125	0.016	0.1286	1.2054 × 10^−4^
#3	0.0278	1.159	0.013	0.1409	1.3124 × 10^−4^
#4	0.0294	1.203	0.012	0.1539	1.3498 × 10^−4^

## Data Availability

All data generated or analyzed during this study are included in this published article.
